# The Integration of Electrical Signals Originating in the Root of Vascular Plants

**DOI:** 10.3389/fpls.2017.02173

**Published:** 2018-01-10

**Authors:** Javier Canales, Carlos Henriquez-Valencia, Sebastian Brauchi

**Affiliations:** ^1^Facultad de Ciencias, Instituto de Bioquimica y Microbiologia, Universidad Austral de Chile, Valdivia, Chile; ^2^Millennium Institute for Integrative Systems and Synthetic Biology, Santiago, Chile; ^3^Facultad de Medicina, Instituto de Fisiologia, Universidad Austral de Chile, Valdivia, Chile; ^4^Millennium Nucleus of Ion Channels-Associated Diseases, Valdivia, Chile

**Keywords:** nutrient transport, action potential, ion channels, apoplast, plasmodesma, sensory epithelia

## Abstract

Plants have developed different signaling systems allowing for the integration of environmental cues to coordinate molecular processes associated to both early development and the physiology of the adult plant. Research on systemic signaling in plants has traditionally focused on the role of phytohormones as long-distance signaling molecules, and more recently the importance of peptides and miRNAs in building up this communication process has also been described. However, it is well-known that plants have the ability to generate different types of long-range electrical signals in response to different stimuli such as light, temperature variations, wounding, salt stress, or gravitropic stimulation. Presently, it is unclear whether short or long-distance electrical communication in plants is linked to nutrient uptake. This review deals with aspects of sensory input in plant roots and the propagation of discrete signals to the plant body. We discuss the physiological role of electrical signaling in nutrient uptake and how nutrient variations may become an electrical signal propagating along the plant.

## The electrical nature of life

Pressure-drive swelling is a problem that emerged early in evolution and solved with the emergence of membrane proteins allowing for the synchronous redistribution of ionic gradients across the plasma membrane. A secondary effect of this solution is the generation a voltage drop within the membrane dielectric (Finkelstein, 1976; Armstrong, 2015). The activity of ion channels and transporters selectively regulates the passage of ions, generating transient local variations in the membrane potential while incorporating metabolites or changing membrane permeability in response to an external signal. Allowing for the synchronization of cellular processes and the communication within cellular communities, electrical sensing, and signaling develops as a wide spread mechanism at the different levels of biological organization. From bacterial biofilms (Strahl and Hamoen, 2010; Masi and Ciszak, 2014; Prindle et al., 2015) to higher plants (Sanderson, 1872; Darwin, 1897; Bose, 1907; Pickard, 1973) and animals (Galvani, 1791; Hodgkin, 1937; Cole and Curtis, 1939; Armstrong, 2007) electrical communication adopt different forms varying in its complexity from simple graduated or oscillating changes in membrane voltage to the long-range electrical signaling observed in excitable cells.

## Electrical signals in animals and higher plants

It is well-established that both plants and animals utilize long-range electrical signaling to transduce environmental information to the whole body (Armstrong, 2007; Hedrich et al., 2016). In multicellular organisms, information must be conducted from detectors to the effector tissue. For the case of animals, the nervous system plays a central role in homeostasis, serving as the primary integrator for most of the relevant physiological information. The communication between epithelial tissue and excitable cells define the way animals interact with the environment, not only by taking advantage of sensory modalities such as touch, temperature, light, or sound (Frings, 2009; Julius and Nathans, 2012) but also by integrating internal processes such as hormonal discharge, gut physiology, and immune system development (Zhang and Zhang, 2009; Bellono et al., 2017; Clemmensen et al., 2017).

Molecular detectors found in sensory epithelia are activated by environmental cues, triggering (directly or indirectly) the opening of an ion channel conductance that changes the local transmembrane potential (Martinac, 2008). In non-excitable cells, such as epithelial cells in the gut or lung, electrogenic transport orchestrates nutrient uptake, controls pH, and modulates water secretion (Boyd, 2008; Beumer and Clevers, 2017; Clemmensen et al., 2017). For the case of epithelia, the absence of suitable voltage-dependent channels (i.e., Ca_v_, Na_v_) impedes the propagation of the initial depolarization over long distances. In excitable cells, depolarization provides the necessary energy to induce the opening of voltage-gated channels (i.e., Ca_v_s, Na_v_s, and K_v_s) (Bezanilla, 2008; Catterall et al., 2017). Propagation speed, the shape of the propagated potential, and the frequency of the electrical message are determined by the cable properties of the cell, which are defined by both the geometry of each particular cell type and the ion channel set available. In animals, this type of communication extends to the multicellular organism when a released substance from a given cell exert an effect in a post synaptic cell (Gerber and Südhof, 2002; Jackson, 2006; Catterall and Few, 2008). As described originally in *Aplysia* by E. Kandel (Castellucci and Kandel, 1976), excitable cells modify their behavior in response to stimulation. Considering that the control of expression, localization, and activity of cellular receptors and ion channels represent the molecular grounding of non-associative learning in animals (Kandel, 2001), it is tempting to question how plants modulate the different ion fluxes and which are the elements conferring plasticity to plant's learning.

Action potentials have been reported in algae and higher plants (Pickard, 1973; Trebacz and Zawadzki, 1985; Kateriya et al., 2004; Fromm and Lautner, 2007; Hegenauer et al., 2016). As foreseen by Davies (1987), nowadays it is widely accepted that electrical signaling plays a major role in inter- and intra-cellular communication of plants. However, in contrast to detailed knowledge of the molecular and cellular mechanisms that governing electrical signaling in animals, the identity of the cellular sensors and effectors, and their exact distribution within the plant, is still unclear (Ward et al., 2009; Hedrich, 2012; Hedrich et al., 2016). Moreover, lacking the sophisticated cellular wiring developed by metazoans to transmit their long-range electrical signals, it seems that plants developed an architecture allowing them to shape—*or forcing them to adapt*—electrical communication differently. Simple questions emerge from this reasoning, how exactly plants wire up? How electrical signals move through the cellular network? How these signals work together connecting environmental sensing, gene expression, nutrient uptake, gas exchange, water balance, energy production, and waste storing? In this review we are not aiming to answer such ambitious questions but rather to put in perspective the different elements that might contribute to the generation and propagation of the electrical message in the root of land plants.

The ability to navigate is an attribute of animals and imposes fundamental problems to solve such as (i) multiplex sensory input at high frequencies, (ii) the rapid integration of these signals, and (iii) to deliver the computed command with exquisite cellular precision, allowing a coherent body response. Unicellular green algae, ancestors of land plants, have navigation capabilities and coincidentally present a different set of ion channels when compared to their descendants, expressing essential elements important in shaping the electrical response of excitable cells in animals (Merchant et al., 2007; Wheeler and Brownlee, 2008). Among these are voltage-activated calcium and sodium channels, TRP channels, and the ryanodine receptor, all absent in modern land plants (Wheeler and Brownlee, 2008; Ward et al., 2009; Fromm and Lautner, 2012; Taylor et al., 2012; Arias-Darraz et al., 2015; Edel et al., 2017). Trapped in the same natural world, animals, and plants share a large set of environmental stress factors. Nevertheless, they have clearly adopted different ways for solving basic problems such as reproduction and self-preservation. Likely the quest for food, mating, and the need for waste disposal cued animals to develop signaling mechanisms that are tuned to navigate. On the other hand, plants not only manufacture their own carbohydrates but also importantly store their waste. Therefore, the sessile nature of land plants demands for robust adaptation mechanisms instead. Accordingly, cellular and molecular sensors are constantly feeding the plant with useful environmental information that has to be distributed through out the body (Karban, 2015). Recent studies suggests that Arabidopsis efficiently organize their three dimensional planning to optimize nutrient supply (Conn et al., 2017), strengthening the idea that plant's architecture is controlled by a management mechanism in charge of the trading between total length of the branches and nutrient distribution. Such mechanism must be associated to the nature and propagation properties of electrical signals generated at the root and leafs, tissues where minerals and water are absorbed, carbohydrates produced, and byproducts stored. Further experimental work on intact living plants, using suitable models allowing for simultaneous electrical and imaging recordings are needed to evaluate the impact of electrical signals on food distribution along the plant (Kanchiswamy et al., 2014; Salvador-Recatalà et al., 2014; Gunsé et al., 2016; Candeo et al., 2017).

### The conducting plant

Missing not only the cellular architecture but also the ion channel set encoding the electrical message in animals (Ward et al., 2009; Hedrich, 2012), there is no reason to suggest that the sensory input in plants is either integrated or processed in a similar way. It has been proposed that the plant phloem forms a single conducting cable, the equivalent of an axon in a single metazoan neuron (Hedrich et al., 2016). Different cell types including companion cells and sieve elements form the phloem. Unlike other plant cell types, sieve elements cells do not present discontinuities in their permeability due to the presence of sieve plates, enabling a continuous transport of solutes between different organs of the plant and providing a low-resistance, high capacitance conduit that allows for the propagation of relatively slow electrical signals. Decades of theoretical and experimental evidence put forward the concept that the phloem would be the principal conduit, able to electrically couple roots and aerial tissues (Brenner et al., 2006; Fromm et al., 2013; Hedrich et al., 2016). Still, the information detected at epidermal cells of the root must propagate through the cortex's cellular network, integrate, and reach the phloem to be transduced all over the plant's body. Conversely, the signal should exit the phloem to have an impact on cells in the aerial tissue. To accomplish this complex task, vascular plants have an inter-connected extracellular space between the plasma membrane and the cell wall (i.e., the apoplastic space) and direct cellular connectivity via plasmodesmata (Sattelmacher and Horst, 2007; Lee, 2015). These peculiarities serve to different signaling functions in the plant, allowing not only the passage of soluble signals but also defining the electrical coupling between cells and the modulation of specific signals associated to the calcium response that comes together with the detection of diverse environmental cues (Zebelo et al., 2012; Nawrath et al., 2013; Lee, 2015; Choi et al., 2016; Edel et al., 2017).

Two major types of long-distance electrical signals have been described in plants, action potentials (APs), and variation potentials (VPs) (Bose, 1907; Pickard, 1973; Fromm and Lautner, 2007, 2012). The former are induced by voltage depolarization, exhibit a threshold potential, follow an all-or-nothing principle, and travel at constant velocity and amplitude, very much like APs observed in the animal kingdom (Zawadzki et al., 1991; Jackson, 2006; Armstrong, 2007; Yang et al., 2016). In contrast, VPs have shown to be induced by a rapid increase in the internal pressure of the xylem, and appear as slow waves of depolarization of variable sizes (Fromm and Lautner, 2007, 2012). A third mode of electrical signal dubbed system potentials (SPs), consisting of hyperpolarization that propagates over medium range distances has also been described (Zimmermann et al., 2009). While VPs depend on the inactivation of P-type H^+^-ATPase, SPs seems to be caused by the activation of the pump. From the early works of Burdon-Sanderson it is known that rise times for plant APs are in the order of about 0.1 s, with durations of about 1 s and rates of propagation of in the order of few hundreds of mm s^−1^ (Sanderson, 1872; Pickard, 1973). These electrical signals not only differ in their shape and magnitude but also in their propagation speed ranging from 1 to 60 mm s^−1^ for APs to several minutes per centimeter in VPs. System potentials are triggered by depolarization, do not have an all-or-nothing character, self-propagate at a constant velocity of about 0.5–2 mm s^−1^, and their magnitude is proportional to the input stimuli (Zimmermann et al., 2009). Interestingly, SPs resemble animal's receptor potentials, self-propagating simultaneously over sensory epithelia. The leaf of arabidopsis, beans, and barley exhibits self-propagating electrical activity, caused by wounding, restricted to leaf-to-leaf communication, and associated to the expression of glutamate receptor-like genes (Zimmermann et al., 2009; Mousavi et al., 2013; Salvador-Recatalà et al., 2014; Salvador-Recatalà, 2016a). In this case, the type of wound seems to be related to distinct types of depolarization. It has been suggested that the anatomy of the tissue will be of importance to define the connectivity between the surface tissue and the phloem (Salvador-Recatalà, 2016a).

Still, it has been difficult to systematize both a theoretical model integrating whole plant electrical signaling and experimental methods to study long-range electrical communication in whole plant configuration (Goldsworthy, 1983; Davies, 1987; Pietruszka et al., 1997; Fromm and Lautner, 2007; Volkov, 2012; Fromm et al., 2013; Hedrich et al., 2016). Nevertheless, it has been established that the different organs of the plant including leaves, stem, flowers, and the root have intrinsic electrical activity (Pickard, 1973; Baldwin et al., 2006; Fromm and Lautner, 2007, 2012; Appel and Cocroft, 2014; Engineer et al., 2015; Karban, 2015; Zhou et al., 2016). Moreover, long-range electrical communication between roots, shoot, and leaves have been described (extensively reviewed in Pickard, 1973; Fromm and Lautner, 2007; Zimmermann et al., 2009; Hedrich et al., 2016). A detailed description of electrical signal transduction on roots is missing, probably due to the seemingly uncoordinated nature of root's APs (Fromm and Eschrich, 1993; Fromm et al., 1997, 2013; Masi et al., 2015; Salvador-Recatalà, 2016b).

## Mapping the ion channel set in arabidopsis

Electrical properties of cells derive from the expression and control of ion channels, transporters, and pumps. These can be modulated by different stimuli such as: pressure, exogenous and endogenous ligands, temperature, light, membrane voltage, and stretch among others. The molecular machinery outlining the propagation of electrical signals in plants is not known in detail but taking into account experimental data and genetic information available we have learned that plants and animals utilize dissimilar strategies to propagate APs. While animals use voltage-sensitive Na^+^ and Ca^2+^ channels to drive depolarization (Hodgkin and Huxley, 1952; Armstrong, 2007; Catterall et al., 2017), the toxic nature of sodium makes plant cells to utilize Cl^−^ and Ca^2+^ instead. While Ca^2+^ will cause depolarization by entering the cell, Cl^−^ will do by leaving the cell. According to gene expression profiles, depolarization of plant cells is likely driven by ALMT/QUAC-type chloride channels and/or ion channels allowing for calcium influx such as two-pore channels (TPCs), cyclic nucleotide-gated channels (CNGCs), or glutamate receptor-like channels (GLRs) (Ward et al., 2009; Hedrich, 2012; Hedrich et al., 2016). In the chain of events defining the AP an initial raise in Ca^2+^ will trigger a Cl^−^ efflux and the subsequent activation of voltage-dependent potassium channels will likely participate in repolarization (Schroeder et al., 1984; Ward et al., 2009; Hedrich et al., 2016).

**Figure 1:**
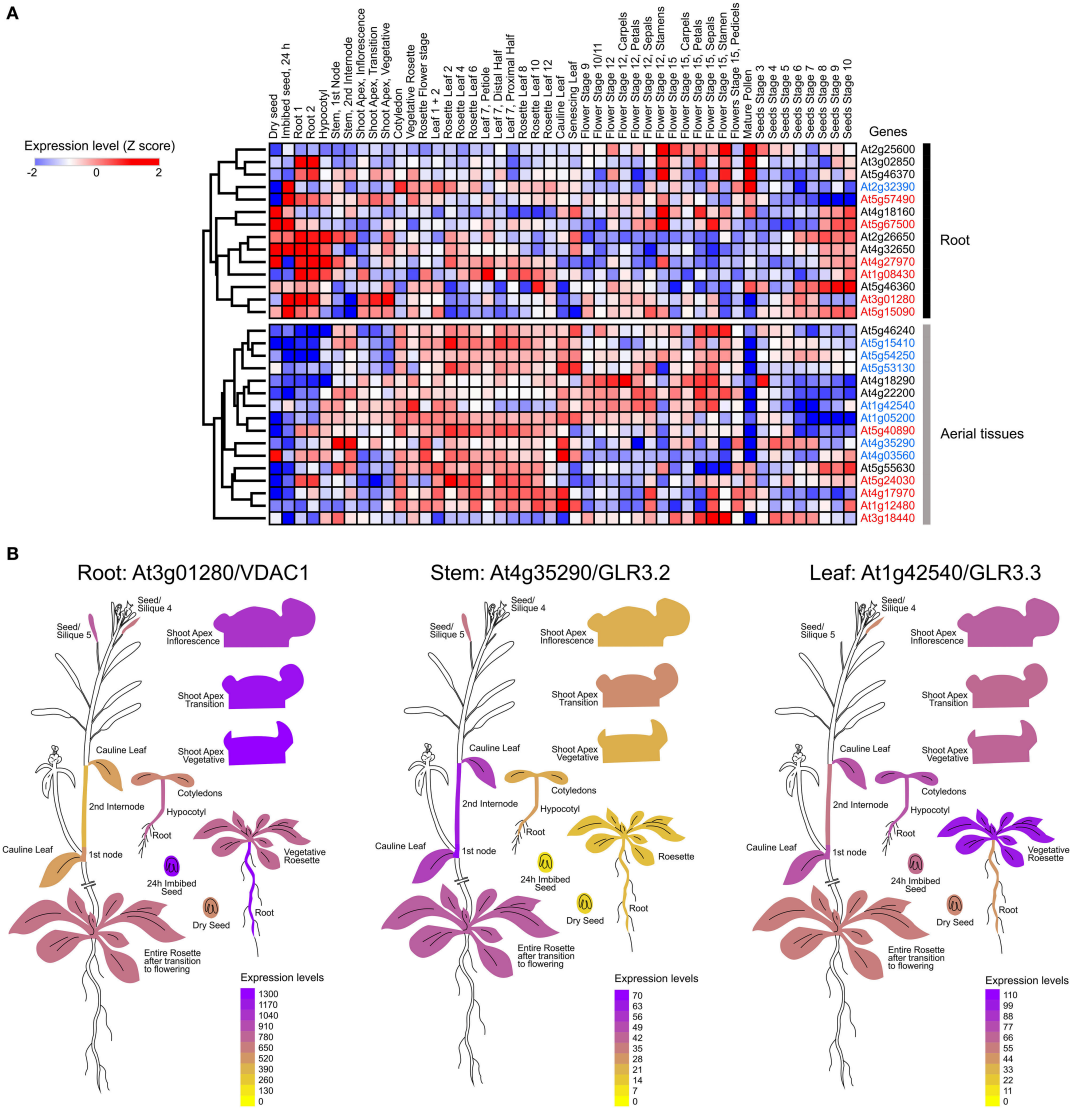
Mapping the expression of ion channels in *Arabidopsis thaliana*. **(A)** Hierarchical clustering of normalized mRNA levels of genes coding potassium (black), calcium (red), and anion (blue) channels. Microarray data were obtained and visualized from the Arabidopsis eFP browser (Waese et al., 2017). Expression values for each gene were transformed to Z-scores across all samples in order to identify tissue-specific expression. High Z-score values (red or blue) indicate a larger deviation from the mean expression across all tissues. **(B)** Examples of ion channels showing tissue-specific expression.

As the ability of a tissue to generate electrical signals will be determined by the ion channel set expressed in the different cell types involved in the passage of the electrical message, we mapped functionally-characterized channels and transporters that have been previously associated to electrical signaling in *Arabidopsis thalina* (Barbier-Brygoo et al., 2011; Hedrich, 2012) (Table 1). We performed a hierarchical clustering analysis to group these genes according to the expression profiles obtained from EPlant (Waese et al., 2017). When comparing all relevant tissues at different stages of development, we observed that the different ion channels present a characteristic pattern of expression (Figure 1A).

**Table 1:** Functional ion channels in Arabidopsis.

	**Name**	**Locus**	**Fuction**	**References**
Voltage-gated K^+^ channel	KAT1	At5g46240	Stomatal opening	Ronzier et al., 2014
	KAT2	At4g18290	Stomatal opening	Ronzier et al., 2014
	AKT1	At2g26650	K^+^ uptake from soil	Xu et al., 2006; Geiger et al., 2009
	SIPK (AKT6)	At2g25600	Pollen tube development	Mouline et al., 2002
	AtKC1	At4g32650	Regulation AKT1	Geiger et al., 2009
	AKT2	At4g22200	K^+^ battery, stomatal movement	Szyroki et al., 2001; Gajdanowicz et al., 2011
	SKOR	At3g02850	K^+^ loading to xilem	Liu et al., 2006
	GORK	At5g32500	Involved in stomatal clousure, stomatal movement	Hosy et al., 2003
Voltage-independent K^+^ channel	TPK1	At5g55630	K^+^ homeostasis, germination, stomatal movement	Gobert et al., 2007
	TPK2	At5g46370	Unknown	Voelker et al., 2006
	TPK3	At4g18160	Unknown	Voelker et al., 2006
	TPK4	At1g02510	K^+^ homeostasis, growing tube pollen	Becker et al., 2004
	KCO3	At5g46360	Unknown	Voelker et al., 2006; Rocchetti et al., 2012
Ca^2+^ channels	CNGC1	At5g53130	Response to pathogen, senescence	Leng et al., 1999; Ma et al., 2010; Chin et al., 2013
	CNGC2	At5g15410	Response to pathogen	Leng et al., 2002; Chin et al., 2013
	CNGC4	At5g54250	Patogen infection	Balagué et al., 2003
	GLR 3.2	At4g35290	Ca^2+^ homeostasis,ionic stress	Kim et al., 2001
	GLR 3.3	At1g42540	Ca^2+^ homeostasis, wound response	Mousavi et al., 2013; Salvador-Recatalà, 2016a
	GLR 3.5	At2g32390	Ca^2+^ homeostasis, wound response	Salvador-Recatalà, 2016a
	GLR 3.6	At3g51480	Ca^2+^ homeostasis, wound response	Mousavi et al., 2013; Salvador-Recatalà, 2016a
	TPC1	At4g03560	Stomatal opening, germination	Peiter et al., 2005; Guo et al., 2016
Voltage-dependent anion channel (VDAC)	VDAC1	At3g01280	Regulate cold stress response, growth pollen	Tateda et al., 2011; Li et al., 2013
	VDAC2	At5g67500	Seedling development, energy production	Yan et al., 2009; Tateda et al., 2011
	VDCA3	At5g15090	Germination, energy production	Tateda et al., 2011; Yang et al., 2011; Berrier et al., 2015
	VDCA4	At5g57490	Energy production, plant growth	Tateda et al., 2011
R-type anion channel	QUAC1 (ALMT12)	At4g17970	Involved in stomatal clousure, stomatal movement, sulfate transporter	Meyer et al., 2010; Malcheska et al., 2017
	AtALMT9	At3g18440	Stomatal opening	De Angeli et al., 2013; Zhang et al., 2014
S-type anion channel	SLAC1	At1g12480	Stomatal opening	Zhang et al., 2016
	SLAH3 (SLAC1 homolog 3)	At5g24030	Stomatal opening, nitrate efflux channel	Zheng et al., 2015; Zhang et al., 2016
Voltage dependent Cl**-**	AtCLCa	At5g40890	NO3- transporter, nitrate homeostasis	De Angeli et al., 2006

**Figure 2:**
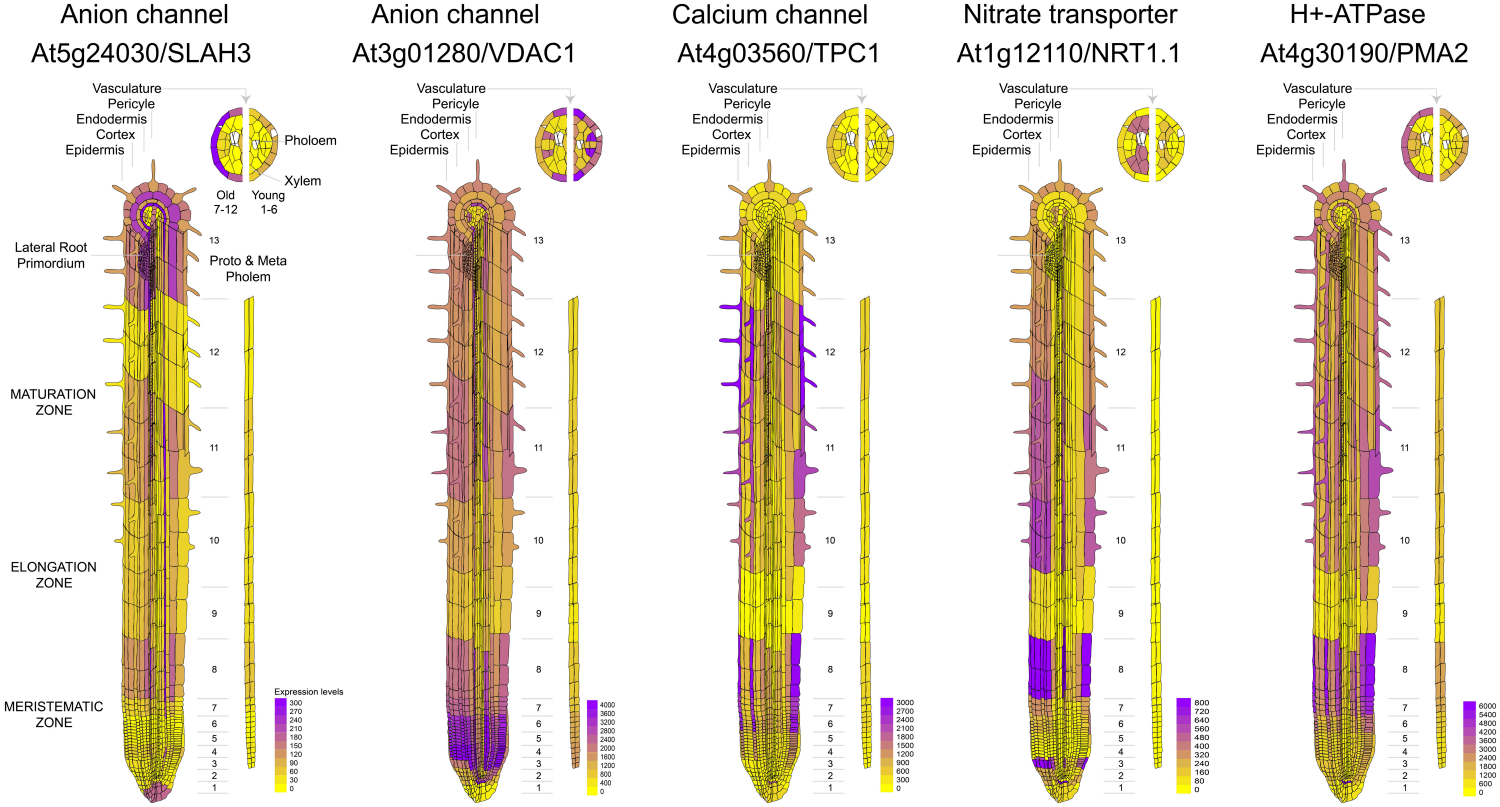
Comparative expression profiles at cell-type resolution of anion channels and genes related to nitrate uptake in the Arabidopsis root. Microarray data was obtained and visualized using the Arabidopsis eFP browser (Waese et al., 2017).

It is known that calcium signaling is important for the control of stomatal opening (Laanemets et al., 2013) and potassium channels are critical in the repolarization phase (Schroeder et al., 1984). As expected, we observed a large expression of GLR and K_v_ channels in the leaves (Figures 1A,B). Although genes encoding for anionic and potassium channels do not show a clear separation between the aerial part and roots (Figure 1A), we found specific genes whose expression is predominant in the roots (VDAC1), stems (GLR3.2), or leaves (GLR3.3) (Figure 1B). These tissue-specific expression profiles suggest that there are different pathways for the generation and propagation of electrical signals in plants and that these routes change during development. When observed in more detail, two root-specific anion channels, SLAH3, and VDAC1, showed different expression pattern across cell types (Figure 2). The voltage-dependent anion channel VDAC1 is strongly expressed along the root tissue. Comparatively, the expression at the meristematic zone is higher than in root hairs (Figure 2). Similarly, H^+^-ATPase is expressed in almost all cell types of the root. In contrast, the slow chloride conductance channel SLAH3 showed greater expression in internal root tissues such as the pericycle and the cortex, important physical barriers on the way to the phloem (Nawrath et al., 2013). While the expression of the electrogenic nitrate transporter NRT1.1 is predominantly observed in root's hairs and at the phloem closer to the stem, the vacuolar channel TPC1 is markedly expressed in root hairs of the maturation zone. Interestingly, none of these membrane proteins showed a marked expression in the phloem along the root (Figure 2).

**Figure 3:**
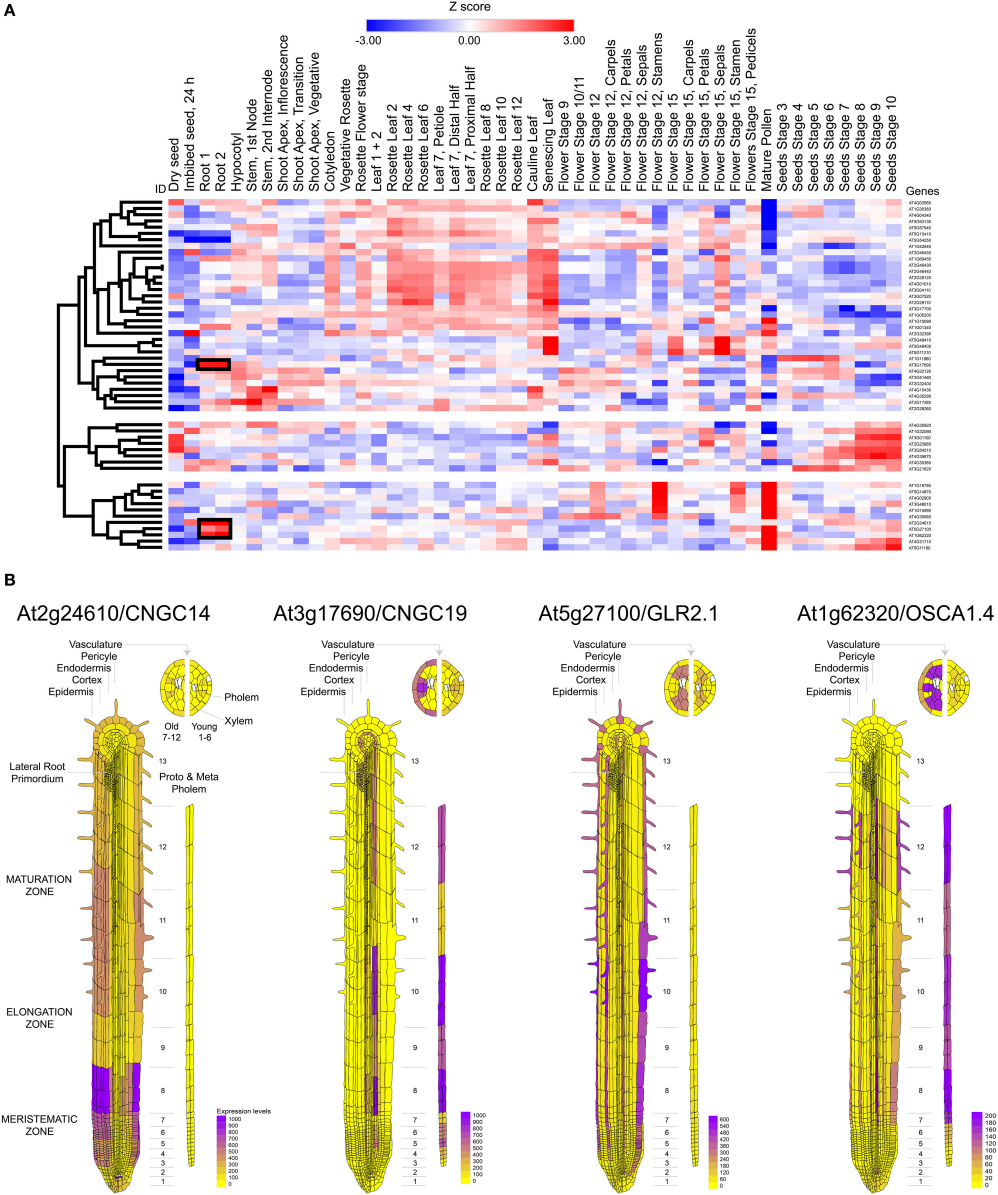
Mapping the expression of calcium channels in *Arabidopsis thaliana*. **(A)** Heat map and clustering dendrograms of the expression of calcium channels among tissue types. Tissue samples are represented in columns and genes in rows. Expression values for each gene were transformed to Z-scores across all samples in order to identify root-specific expression, which is indicated with black rectangles. **(B)** Calcium channels showing root-specific expression.

Calcium influx in response to external stimuli seems critical for the generation of the electrical signal. Nearly 50 different ion channels have been associated to Ca^2+^ influx in land plants. This large set of calcium channels are segregated in five different families: CNGC, GLR, TPC1, osmotic response-related channels (OSCA), and mechano-sensitive calcium channels (MCA) (Kurusu et al., 2012; Chin et al., 2013; Morgan and Galione, 2014; Edel et al., 2017) (Table 2). From these channels CNGC14, CNGC19, GluR2.1, and OSCA1.4 appear to be preferentially expressed in the root tissue (Figure 3A). The expression of these channels was also observed to be differential. CNGC14 is largely expressed at the epithelium close to the meristematic zone and to a lesser extent at the maturation zone. In contrast, the vacuolar channel CNGC19 is concentrated at the endothelium and the phloem. On the other hand, the ligand gated GLR2.1 and osmotic-related OSCA1.4 channels are preferentially expressed in root hairs. While the expression of GLR2.1 at the epithelial tissue somewhat decreases from the meristematic zone toward the maturation zone, OSCA1.4 is preferentially expressed at the maturation zone and the phloem (Figure 3B).

**Table 2:** Calcium channels in Arabidopsis.

**Channel family**	**Name**	**Locus**
CGNCs	CNGC10	AT1G01340
	CNGC7	AT1G15990
	CNGC8	AT1G19780
	CNGC6	AT2G23980
	CNGC14	AT2G24610
	CNGC15	AT2G28260
	CNGC3	AT2G46430
	CNGC12	AT2G46450
	CNGC19	AT3G17690
	CNGC16	AT3G48010
	CNGC13	AT4G01010
	CNGC17	AT4G30360
	CNGC9	AT4G30560
	CNGC18	AT5G14870
	CNGC2	AT5G15410
	CNGC1	AT5G53130
	CNGC4	AT5G54250
	CNGC5	AT5G57940
GLRs	GLR3.4	AT1G05200
	GLR3.3	AT1G42540
	GLR3.1	AT2G17260
	GLR2.3	AT2G24710
	GLR2.2	AT2G24720
	GLR2.9	AT2G29100
	GLR2.8	AT2G29110
	GLR2.7	AT2G29120
	GLR3.5	AT2G32390
	GLR3.7	AT2G32400
	GLR1.1	AT3G04110
	GLR1.4	AT3G07520
	GLR3.6	AT3G51480
	GLR2.4	AT4G31710
	GLR2	AT4G35290
	GLR2.6	AT5G11180
	GLR2.5	AT5G11210
	GLR2.1	AT5G27100
	GLR1.2	AT5G48400
	GLR3.1	AT5G48410
TPC	TPC1	AT4G03560
MCAs	MCA2	AT2G17780
	MCA1	AT4G35920
OSCAs	OSCA2.2	At1g10090
	OSCA1.3	At1g11960
	OSCA3.1	At1g30360
	OSCA1.8	At1g32090
	OSCA2.1	At1g58520
	OSCA1.4	At1g62320
	OSCA2.4	At1g69450
	OSCA2.3	At3g01100
	OSCA1.5	At3g21620
	OSCA2.4	At3g54510
	OSCA1.7	At4g02900
	OSCA1.1	AT4G04340
	OSCA1.6	At4g15430
	OSCA1.2	At4g22120
	OSCA4.1	At4g35870

**Figure 4:**
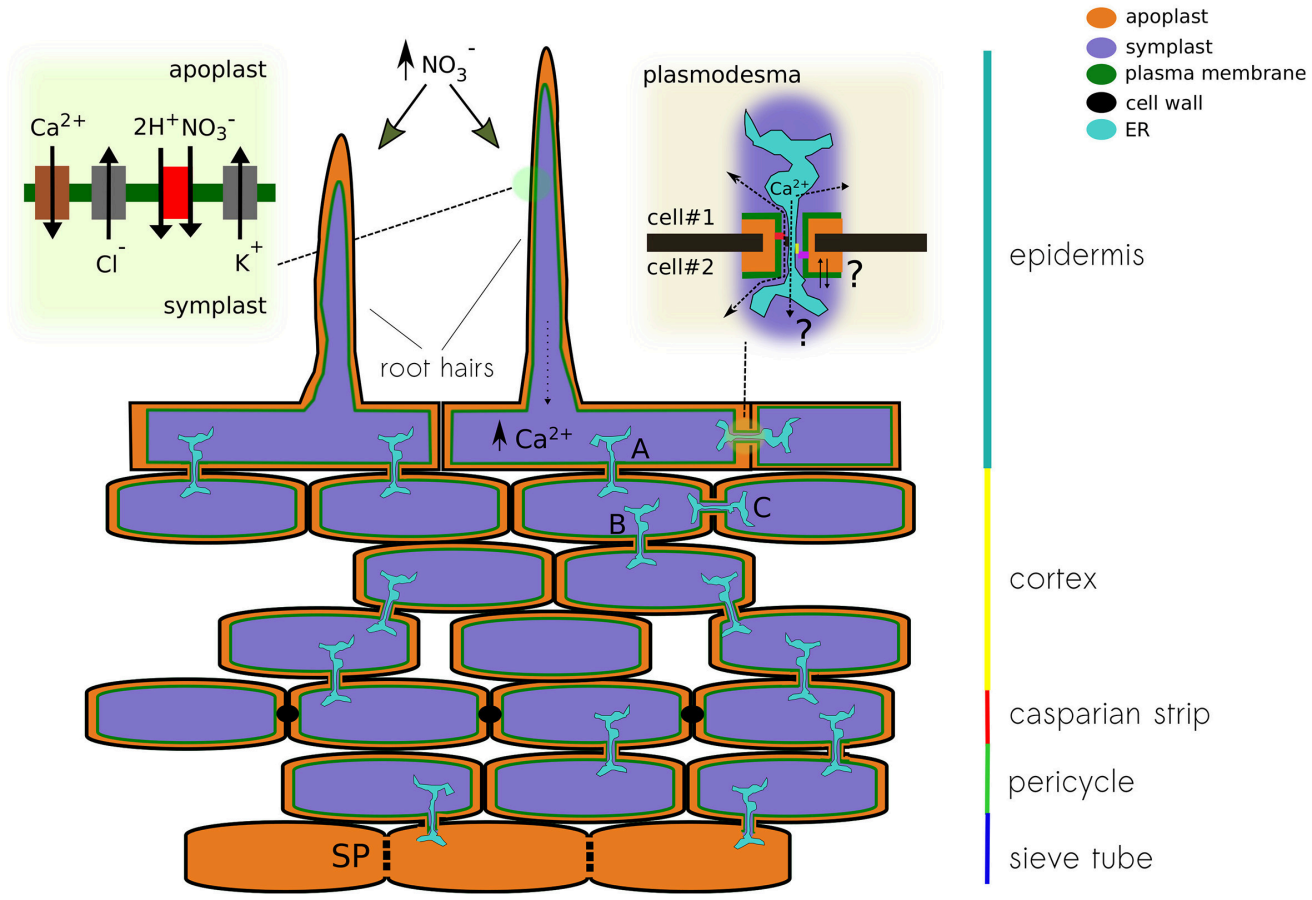
Schematic representation of a plant root showing different pathways for the propagation of electrical signals. The different conducting state of plasmodesma A, B, and C will modulate the signal detected at the root hair on its way to the sieve tube. The upper left panel show the conductances that might be associated to nitrate-induced depolarization. Calcium increase in the epithelial cell modulates the activity of the plasmodesma. Both calcium levels and physical changes of the plasmodesma might modulate the activity of ion channels expressed in that region. The upper right panel pictures the cytoplasmic and reticular pathways for ions, both can be modulated by the interaction with anchoring proteins of the contact site of the pore.

Nitrate treatments in nitrogen-starved plants induce a transient depolarization of the plasma membrane (Meharg and Blatt, 1995; Wang and Crawford, 1996; Wang et al., 1998). Likewise, it has been recently reported that nitrate treatments trigger an intracellular calcium increase, which initiates the nitrate-signaling pathway (Liu et al., 2017). Moreover, genetic evidence indicates that elevations in intracellular calcium are associated to NRT1.1. (Riveras et al., 2015). Given the observed expression profile, we may hypothesize that a nitrate uptake-induced depolarization of the epithelial cell, caused by an increase in the activity of the nitrate transporter NRT1.1, will trigger calcium influx through OSCA1.4 and/or GLR2.1, further activating a calcium or voltage-dependent chloride conductance (e.g., SLAH3 or VDAC1), allowing for the propagation of the electrical signal along the cortex toward the phloem (Figure 4).

## Cellular connectivity as part of plant's electrical signaling

A united model connecting plant sensing and controlled behavior is needed to explain whether the stimuli detected at the boundaries of the plant body (e.g., root hairs, leafs' epithelial cells), transduce the electrical information to the phloem cable, how the signal is further integrated, and lastly how the electrical signal exits the phloem, reaching the effector tissue located in a distant epithelia (e.g., guard cells at leaf stoma). While the differential expression of the ion channel set is important to determine excitability, the plant's interconnected cellular architecture will be critical in governing the amplitude and propagation properties in three-dimensional space. Modulation of these elements in response to repetitive, acute, or chronic sensory input may confer plasticity to the plant's response and will allow for both learning and adaptation, without the need for a “brain-like” integrator or cognitive behavior as suggested in literature (Baluska et al., 2004).

One of the main structural differences between animal and plant cells is the presence of a cell wall. The plant cell wall creates an unusual extracellular environment known as the apoplast which constitutes a physical/chemical barrier that participates in cell-to-cell communication pathways including long-range electrical signaling (Sattelmacher, 2001; Fromm and Lautner, 2007; Choi et al., 2017). In plants, the extracellular concentration of ions corresponds to the apoplastic ionic concentrations, which are highly regulated. The ionic composition of the apoplast is variable and depends on both internal factors such as tissue or development stage and external factors such as nutritional stress (López-Millán et al., 2001). Ions in the apoplastic compartment are usually found in low concentrations consisting predominantly of inorganic cations and anions such as K^+^, Ca^2+^, Mg^2+^, Cl^−^, ${\text{NO}}_{3}^{-}$, and ${\text{PO}}_{4}^{3-}$ (Gabriel and Kesselmeier, 1999). The apoplastic and intracellular ion concentrations together with ion permeability will define the resting potential of the root epidermal cell and has been reported to be about −120 mV, negative inside (Fromm and Eschrich, 1993). Biochemical properties of the cell wall will define the apoplastic space and nutrient permeability in the first place. In the adult plant, a high content of lignin or suberin in cell walls considerably decreases their permeability (Nawrath et al., 2013) and therefore constitutes a tight barrier to free diffusion of nutrients in and out of the plant tissue. In contrast, the structure and composition of cell walls change during root development (Somssich et al., 2016). The meristematic zone contains young cells localized close to the root tip, while the older cells are localized at the root base close to the stem. Cell walls of the meristematic zone are thin and more permeable because of a higher mitotic activity (Baluska et al., 1996). Cells from the differentiation zone are more rigid due to the accumulation of lignin associated with the development of secondary cell walls, which provides extra strength to the walls and makes them waterproof Somssich et al. (2016). Therefore, there is a longitudinal permeability gradient in primary roots determined by the differentiation degree of epidermal cells.

The equilibrium concentration of ions can be calculated from the Nernst equation of the form [X]_out_/[X]_in_ = exp(V_m_ZF/RT), were [X]_out_ and [X]_in_ are the external and internal concentrations of an ion X, V_m_ is the membrane voltage, Z is the valence of the permeable ion, F the Faraday constant, R the gas constant, and T the absolute temperature. From this equation, it is clear that variations in the extracellular concentration of a permeable charged solute will affect the membrane potential at rest. In fact, early studies have shown that both the peak of the action potential and magnitude of the inward current are dependent on the apoplastic calcium concentration (Hope, 1961a,b; Hope and Findlay, 1964). On the other hand, the activity of the H^+^-ATPase maintains apoplastic pH usually acidic (4.7–5) (Sattelmacher and Horst, 2007). As the resting membrane potential in plant cells is likely to be set by proton transport, therefore, one should expect a high sensitivity to variations in the activity of the proton pump (Brault et al., 2004). Considering that the apoplast occupies a relatively small volume of the plant tissues, representing less than 5% for the case of leaves (López-Millán et al., 2001) it is reasonable to expect that environmental changes might cause rapid alkalization of the apoplast that in turn will produce a local depolarization (Grams et al., 2009). Anionic nutrients such as nitrate, sulfate and phosphate are acquired passively by means of electrogenic co-transport helped by the proton gradient (Ullrich-Eberius et al., 1981; Muchhal and Raghothama, 1999). Likewise, apoplastic pH will be sensitive to the activity of phosphate and nitrate transporters (Amtmann et al., 1999). Accordingly, acute treatments with nitrate in nitrogen-starved plants induce a transient depolarization of the plasma membrane (Meharg and Blatt, 1995; Wang and Crawford, 1996). Experimental evidence suggests that 2–4 protons are co-transported during phosphate uptake (Sakano, 1990). For the case of nitrate, the stoichiometry of co-transport with protons has been calculated to be 2:1 (McClure et al., 1990; Glass et al., 1992; Wang and Crawford, 1996). Considering experimental evidence from *in-vitro* studies with membrane vesicles and also genetic analyses such as yeast complementation, the most probable stoichiometry for proton/sulfate co-transport would be 3:1 (Buchner et al., 2004).

The root is the first organ that comes in contact with water and nutrients. Therefore, when plants are deprived of nutrients, the root constitutes a primary site for detection. Comparative transcriptomic analyses between root and shoot samples showed that the plant's response to nitrate initiates at the root (Wang et al., 2003). Plants must integrate the information from the external environment and contrast it with their nutritional status. In fact, it has been reported that the balance between nitrogen and carbon is important for the control of nitrogen assimilation (Zheng, 2009). Therefore, plants require an efficient communication system between the site of nutrient perception and uptake (i.e., roots) and the metabolic center where carbon is produced (i.e., leaves) to respond adequately to changes in nutrient availability. Epidermal cells of the plant root elicit a higher permeability through a cell wall, making electrogenic transporters at the plasma membrane to face high concentrations of nutrients when they happen to get dissolved in the soil surrounding the root. The uptake of any these nutrients will produce a rapid and transient membrane depolarization of the epithelial cell in the root (Dunlop and Gradiner, 1993; Meharg and Blatt, 1995). Moreover, the diffusion of substances within the root's apoplast is internally restricted by the Casparian strip, a lignin-made hydrophobic impregnation of the primary cell wall that seal the extracellular space of endodermal cells, forcing the passage of ions, nutrients, and water through the plasma membrane of endothelial cells (Nawrath et al., 2013; von Wangenheim et al., 2017) (Figure 4).

In addition to the apoplastic communication pathway in the extracellular space, the cytoplasm of plant cells can be internally connected by cell-to-cell junctions known as plasmodesma (Lee, 2015; Kitagawa and Jackson, 2017). Intercellular communication of root tissues has been demonstrated by the rapid diffusion of fluorescent tracer molecules (e.g., propidium iodide) through plasmodesmata into inner layers of the root tissue (Nawrath et al., 2013). It has been demonstrated that the undifferentiated cells from the apical root meristem and elongation zone are dye-coupled and, therefore, are symplastically connected through plasmodesmata (Duckett et al., 1994). Ions and larger molecules can freely diffuse though the pore from one cell to the other making these cellular structures a focal point of signaling through the cortex tissue (Burch-Smith and Zambryski, 2012; Lee, 2015). Moreover, the complexity of plasmodesmata is underscored by the presence of endoplasmic reticulum (ER) passing through the pore, providing a secondary and likely more selective pathway of communication between neighbor cells. It is known that different lipids and *callose*, a soluble protein able to occlude the cytoplasmic pathway, tune permeation through plasmodesmata (Tilsner et al., 2016). Moreover, it has been reported that cytoplasmic calcium elevations promote the closure of the cytoplasmic pathway of the pore (Lee, 2015). Additional data is needed to determine the contribution of these ER tubes in the propagation of both calcium and electrical signals through root cortex.

In plants, direct coupling between the ER and the electrical activity at the plasma membrane is not associated to the stromal interaction molecule 1 (STIM1) or to Orai1 calcium channels, as in animal cells. STIM-related proteins were lost at the level of single-celled algae and Orai relatives are present only up to gymnosperms (Edel et al., 2017). Nevertheless, it has been suggested that anchoring proteins, cytoskeleton elements, and lipids might modulate localization and activity of membrane proteins at the contact site between ER and plasma membrane (Lee, 2015). Thus, the differential expression and modulation of a specific set of calcium-sensitive ion channels, plasmodesmata occlusion, or the remodeling of the pore's shape at the contact site might provide amplification or suppression of the propagating electrical signal through the cortex (Figure 4).

The differentiation of root epidermal cells in Arabidopsis progressively reduces these cytoplasmic connections in such a way that become symplastically uncoupled in the last stage of their development (Duckett et al., 1994). Conversely, the cells of the hypocotyl epidermis are symplastically connected to one another regardless of their state of development (Duckett et al., 1994). Earlier evidence of electrical coupling was given by Spanswick and Costerton, who showed that when injecting current in a cell of the multicellular alga Nitella, the signal could be traced several cells away from the site of injection (Spanswick and Costerton, 1967).

Recent calcium imaging experiments using a genetically encoded ratiometric calcium indicator (i.e., Y-Cameleon 3.6), expressed in living Arabidopsis, showed how the spontaneous response originating in a root hair propagates through the root tissue in well-defined “patches” (Candeo et al., 2017). Although the authors did not comment about this patterned response, by analogy to sensory receptors in animals, it is tempting to interpret such readout as the physical dimension of the “*sensory field*” that correspond to a particular epithelial cell or a group of them. After the stimulation of a cell from root epidermis, an electrical signal is generated; in the example above, the influx of calcium can be directly related to cell depolarization. The generated electrical signal can be transmitted via plasmodesmata to neighbor connected cells in the root cortex. Once the signal reaches the low-resistance sieve tube in the phloem, it propagates throughout the entire plant (Figure 4). The question then is whether the phloem integrates multiple signals coming from individual receptor fields dispersed along the root's epithelia and how the network of cell-to-cell connectivity might provide plasticity, modulating the propagation of the electrical message by controlling the localization and activity of plasmodesmata (Figure 4). Moreover, diffusion experiments with fluorescent dyes showed that the communication of the hypocotyl epidermal cells ends at the base of the stem (Duckett et al., 1994). Therefore, it is likely that epidermal cells of the hypocotyl and root are electrically uncoupled, making sieve tubes the only pathway possible to propagate APs from the root to the shoot. All these physical barriers create nodes of resistive elements, useful for signal filtering not only at the exit of root tissue but also in and out of branches coming out of the stem.

It has been proposed that plants might integrate information through a “*brain-like*” structure, located in the root apex (Baluska et al., 2004; Brenner et al., 2006). However, the striking features of animal's brain not only come from the fact that neurons establish synapses, but rather from the ability to create complex cell-based logic circuits. A single neuron can host hundreds of cell-specific synapses, each one capable of plastic adaptation. In the absence of this kind of cellular interaction, it sounds unlikely that the integration of electrical information in the whole plant led to cognitive processing of sensory information. Moreover, recent works on unicellular organisms such as slime mold and bacteria demonstrate that there is no need for a central nervous system to spawn intelligent solutions (Nakagaki, 2001; Kotula et al., 2014).

Making a very simplistic picture of plant electrical connectivity, we observe it composed by a very inefficient cable surrounded by epithelial tissue formed by absorptive and excitable cells. In such a model epithelial hair cells of the root might function as spines in a neuron, providing signals generated by external stimuli to the cortex circuit where they are processed before reaching the phloem. At the same time, the transition from root to the stem might provide an additional filter, acting as a macroscopic version of the spine neck modulating the transit of long-range electrical signals. The number of cells in the cortex that are electrically connected define the size of the circuit's capacitor and outlines the electrical properties of the paths reaching the phloem. The presence of hypothetical “synapse-like” structures based on actin might contribute in shaping the network of cell-to-cell connectivity (Baluška et al., 2005). In this context, systemic soluble signals such as auxins might also play an important role in regulating cell-to-cell connectivity, for example by interacting with plasmodesmata (Baluska et al., 2004; Brenner et al., 2006). Furthermore, sensory input might also affect fundamental cable properties along the phloem conduit, shaping and filtering the propagated action potentials. The nature of the physical/molecular barriers (e.g., root cortex-to-phloem and phloem-to-leaf epithelia transitions) will be of mayor importance to understand the integration of sensory information in plants.

## Nutrient sensing and long-range electrical signaling

Plants acquire the essential chemical components for the synthesis of biomolecules from the minerals present in the soil (Maathuis, 2009). Phosphorus, nitrogen, sulfur, potassium, magnesium, and calcium are nutrients required in greater quantities by higher plants (Kirkby, 2011). Therefore, plants must acquire these nutrients continuously to ensure suitable growth and development (Maathuis, 2009). Unlike heterotrophic organisms, plants mainly acquired nutrients in inorganic form by specific transporters localized in the roots. The expression of these nutrient transporters, as well as their activity, is regulated by nutrient availability, metabolism and environmental factors (Giehl and von Wirén, 2014). Lacking one of these essential nutrients has a direct impact on plant growth and development, especially in the case of root tissue (Gruber et al., 2013). Consequently, plants have developed sophisticated regulatory systems to ensure the uptake of these inorganic nutrients (Schachtman and Shin, 2007). In fact, the response to nutrient starvation involves complex signaling networks including sensor proteins (Ho et al., 2009), transcription factors (Rubio et al., 2001), miRNAs (Vidal et al., 2010), peptides (Ohkubo et al., 2017), and phytohormones (Kiba et al., 2011). Interestingly, these signaling pathways not only trigger short-term responses involving metabolic adjustments and/or regulation of nutrient transporters, but also induce modifications of the root system architecture.

Among macronutrients, phosphorus and nitrogen have a greater impact on the root architecture when compared to others (Gruber et al., 2013). Phosphate starvation strongly induces the development of root hairs (Péret et al., 2011), whereas the addition of nitrate causes an increase in root hair density (Canales et al., 2017). In addition, phosphate deficiency causes an important decrease of primary root growth and stimulates the development of lateral roots (Shahzad and Amtmann, 2017). In contrast, lower availability of nitrate increases the primary root growth and decreases the development of lateral roots. These opposite effects on the cellular architecture of the root suggest the presence of different signaling pathways for nitrate and phosphate, probably associated to electrical signals of different nature that do not necessarily propagates in the same way or generate in the same epithelial cell type.

## Conclusion

Intracellular calcium variations in root hair cells of plant epidermis are generated by a mechanism involving a local variation in membrane potential, caused by electrogenic transport or by the direct activation of plant receptors by extracellular ligands. These local variations in membrane voltage will provide amplification of the input signal by increasing calcium permeability of the epithelial cell and further trigger action potentials by increasing the permeability of chloride conductances, followed by potassium/proton-driven repolarization. These electrical signals are initially propagated through cells in the cortex by highly regulated networks of plasmodesmata, and by taking advantage of the continuum of the apoplastic space until reaching the sieve tube of the phloem, where it get access to be transmitted throughout the plant body. Physical barriers, ion channel distribution, and cell-to-cell communication in the root are critical aspects that shape the electrical signals generated at the sensory tissue. All subject of cellular control, these elements might provide plasticity to plant response. The role of plasmodesmata in the propagation and fine-tuning of electrical signals in plant's sensory epithelia is a fundamental topic somewhat neglected and evidently requires more attention.

## Author contributions

SB, JC, and CH-V wrote the paper. SB and JC prepared figures.

### Conflict of interest statement

The authors declare that the research was conducted in the absence of any commercial or financial relationships that could be construed as a potential conflict of interest.
